# 

**Published:** 1883-08

**Authors:** 


CONTENTS:

Art. I.—The Odontological Society of Great Britain....145

Art. II.—Zinc Dies for Swaging Plates. By Wm. H. Dorrance, D. D. S....162

Art. III.—On the Density of Enamel as an Element in Determining the Proper Time to Devitalize the Tooth Pulp. By J. Edw. Bine, D. D. S., Rochester, N. Y....170

Art. IV.—Exposed Pulp and Treatment. By Dr. C. R. Rowley, Niles, Mich....175

Art. V.—A Consideration of Some of the Causes of Dental Decay. (Continued). By Alfred Carpenter, M. D....178

Art. VI.—Remedy For Inflamed and Sore Mouths, Caused by Wearing Artificial Teeth on Rubber Plates. By D. G. Harkins, Holyoke, Mass....180

Art. VII.—Calcareous Deposits. By L. C. Ingersoll, D. D. S., Keokuk, Iowa....182

MONTHLY SUMMARY.

Things to be Considered in Regulating Teeth....184
Be Tolerant....186
Tempering Steel....187
Mankind's Mistakes....187
A Rare Case of Exostosis....188
The Effect of Nitrate of Silver on the Skin and Mucous Membrane....189
Ear-Ache in Children....189
Hardening Steel....190
Gluten....190
Practical Suggestions During Dentition....190

